# Pediatric Intensive Care Provider Attitudes About Children with Medical Complexity and Neurologic Impairment: A Qualitative Study

**DOI:** 10.3390/children12010034

**Published:** 2024-12-28

**Authors:** Elizabeth J. Bleed, Leonardo Barrera, Mickayla Jones, Seema K. Shah, Megan Crowley-Matoka, Carolyn C. Foster

**Affiliations:** 1 Ann and Robert H. Lurie Children’s Hospital, 225 E. Chicago Ave, Chicago, IL 60611, USA; lbarrera@luriechildrens.org (L.B.); mijones@luriechildrens.org (M.J.); seema.shah@northwestern.edu (S.K.S.); ccfoster@luriechildrens.org (C.C.F.); 2 Division of Pediatric Critical Care, Department of Pediatrics, Northwestern University Feinberg School of Medicine, 225 E. Chicago Ave #73, Chicago, IL 60611, USA; 3 Division of Advanced General Pediatrics and Primary Care, Department of Pediatrics, Northwestern University Feinberg School of Medicine, 225 E. Chicago Ave #162, Chicago, IL 60611, USA; 4 Medical Education, Northwestern University Feinberg School of Medicine, 420 E. Superior St., Chicago, IL 60611, USA; m-crowley-matoka@northwestern.edu; 5 Anthropology, Northwestern University, 1810 Hinman Ave, Evanston, IL 60208, USA

**Keywords:** attitude of health personnel, chronic disease, disabled children, intensive care units, pediatric

## Abstract

(1) Background: Children with medical complexity (CMC) and neurologic impairment (NI) are a growing population in pediatric intensive care units (PICUs). (2) Objective: Our aim was to explore and describe the experiences and beliefs of PICU providers caring for CMC with NI. (3) Methods: A qualitative interview-based study was conducted. Participants were 20 providers (12 attendings and 8 nurse practitioners) who met inclusion criteria of being a faculty, fellow, or advanced practice provider who worked in a PICU; residents were excluded. Participants were recruited via purposive and snowball sampling until information power was reached, and came from seven PICUs across six states, with 10 participants from the authors’ home institution and 10 from external PICUs. Data were collected via recorded videoconference interviews, which were transcribed. Analysis was conducted and relevant themes were identified using the analytic technique of thematic analysis. Rigor was assured by using two coders. (4) Results: Four main themes were identified: (i) providers view CMC with NI as a distinct population of growing importance; (ii) CMC with NI have care needs that challenge traditional perceptions of PICU practice; (iii) PICU providers expressed ambivalence towards caring for CMC with NI; and (iv) some PICU providers have developed adaptive strategies. (5) Conclusions: This population challenges the typical notion of what pediatric critical care represents. Providers display ambivalence about caring for these patients but can develop strategies to make this work meaningful. Understanding PICU clinicians’ views about CMC with NI can provide insights for improved patient care and reduced provider burnout as the field adapts to this population.

## 1. Introduction

Children with medical complexity (CMC) make up an increasing proportion of hospitalized children [1]. They also represent about half of all pediatric intensive care unit (PICU) admissions, and three-quarters or more of critical care procedures and interventions [2]. CMC tend to die in hospitals, and about a third of CMC have two or more markers of intensity (ICU admission, intubation, dialysis, etc.) in the last month of their lives [3]. They are at higher risk of adverse events and central-line-associated bloodstream infections [4,5]. Chronic neurologic impairment (NI) is not synonymous with medical complexity but frequently co-occurs. Over half of CMC followed by complex care clinics have NI [6], and the majority of children with NI are or become medically complex, accruing multiple chronic conditions in other organ systems in the first several years of life [7]. CMC with NI can be understood as a subset of children with disabilities [8,9]. There are widespread biases against people with disabilities in society generally, and specifically among healthcare providers [8,10,11,12]. Qualitative work suggests patients’ pre-existing complex conditions or disabilities may affect pediatric providers’ medical decision making about life sustaining therapies [13,14]. A recent highly publicized study by Ames et al. described in detail the disability-related biases experienced by families of CMC from their healthcare providers [8].

Previous qualitative work has described the experiences of the families of CMC during PICU admissions [15,16], and the perspectives of PICU nurses and respiratory therapists caring for this population [17,18]. A series of important qualitative studies have examined provider experiences caring for chronically critically ill (CCI) children; they describe a mismatch between these patients and the ICU environment, and identify them as a source of conflict, stress, distress, and burnout for clinicians. These studies included PICU providers in a mixed cohort of either families, non-PICU doctors (i.e., outpatient, palliative care), or non-provider PICU staff [19,20,21,22,23]. Importantly, none of the studies attended specifically to the neurodevelopmental or disability status of the patients.

Because of the growing importance of CMC in the PICU context, existing data showing that healthcare providers may have biases when it comes to patients with disabilities, and the strong co-occurrence of NI and medical complexity, it is important to specifically examine the attitudes of PICU providers towards their patients with both CMC and NI.

## 2. Materials and Methods

### 2.1. Study Overview, Design, and Objectives

A qualitative, interview-based study was designed which aimed to explore and describe the experiences and beliefs of PICU providers caring for CMC with NI to identify the ways that these experiences and beliefs may impact providers’ perspectives of care delivery to this population. A qualitative rather than quantitative approach was chosen because it allowed us to capture the nuanced emotions, attitudes, and stories that may influence provider beliefs and experiences.

### 2.2. Participants, Sampling, and Inclusion and Exclusion Criteria

Inclusion criteria: Eligible PICU providers who were attending pediatric intensivists, PICU fellows, pediatric hospitalists working in PICUs, or advanced practice providers (APPs—inclusive of nurse practitioners (NPs) and physician assistant/associates) working in PICUs. Exclusion criteria: Residents were excluded since their experience in the PICU is relatively limited. Bedside team members (i.e., nurses and respiratory therapists) and family members were excluded to permit a narrow focus on the prescribing provider experience and because bedside and family experiences have been described elsewhere [15,16,17,18,19,22]. Purposive sampling was used to identify participants through professional networks (via email), postings on relevant listservs, and snowball sampling. An estimated need for 20 providers was based on experienced team members’ judgment and it was confirmed during analysis that information power was achieved [24]. An attempt was made to balance the sample between internal and external providers (relative to the researchers’ home institution) but we did not specifically include or exclude providers or PICUs based on specific criteria.

### 2.3. Data Collection

Each provider participated individually in a single semi-structured interview conducted over Zoom (Zoom Video Communications, Inc, Version 5.14.718149) in the Spring–Fall of 2021. A semi-structured interview guide (File S2) was developed based on the specific aims of the study and was piloted with a local PICU provider. The interview guide was iteratively refined during the study period (Supplementary Material). Providers were told the purpose of the study was to ultimately improve the quality of care of CMC in the PICU as well as improve the experiences of providers caring for them. Providers were read definitions of CMC according to Cohen et al. [25] and NI according to Berry et al. [6] to clarify the population of interest. Interviews were recorded and transcribed verbatim and de-identified and lasted 45–60 min; no field notes were collected and repeat interviews were not necessary. Providers also completed a short demographic survey (type of provider, race, years of experience, and presence of a complex care program at their institution) via REDCap electronic data capture tools hosted by Northwestern University [26,27].

### 2.4. Data Analysis

Provider interview transcripts were deductively analyzed by the principal investigator and study team members using qualitative analysis software (Dedoose Version 9.0.107 (2024)) [28] The interview guide and two provider transcripts were initially reviewed and discussed by team members (EB, MJ, and LB) to create an initial codebook. After the codebook was created and refined, each transcript was separately coded by team members (EB or LB), and they reviewed the other’s coding for each transcript to establish agreement, ensure reliability, and refine code definitions. Once agreement was established for all transcripts, all code excerpts were extracted and reviewed to identify themes, using the descriptive and interpretive technique of thematic analysis, similar to that described by Braun and Clarke [29]. Team members (EB and LB) each reviewed a portion of the content of extracted codes in different theme subsets and pooled their findings. This article focuses on the subset of themes related to provider perception of their work in critical care and their mixed emotions about CMC with NI.

### 2.5. Methodological Rigor, Research Team, and Reflexivity

Credibility was established through several methods [30]. Informal member checking was achieved via EB’s regular research presentations to her division of pediatric critical care medicine during the years the research was being conducted. Having LB and EB each analyze parts of the data and having the entire research team discuss themes, analysis, and interpretation served as a form of analyst triangulation. Source triangulation was attained by having participants from both internal and external institutions and from several different institutions. Confirmability was established through analyst triangulation as described above, and through attention to reflexivity. A strength of this team was its composure of clinical and non-clinical researchers with backgrounds in various disciplines including law, anthropology, other qualitative methods, and primary care. EB (she/her) was the principal investigator (PI) for this study, with team members LB (he/him) and MJ (she/her) also conducting data collection and analysis. At the time of the study, EB was a clinical PICU fellow and LB and MJ were staff researchers at Lurie Children’s Hospital. Both LB and MJ have prior experience conducting qualitative research. EB acknowledges her own clinical experience caring for CMC with NI and career goal of advocating for and improving the care of this population; having the perspectives of a team of non-PICU researchers helped balance this. The PI (EB) conducted all interviews with providers from external institutions. The internal cohort of participants was at risk of bias if a current trainee interviewed her colleagues. Therefore, a second researcher (MJ, an internal researcher not acquainted with members of the PICU staff) conducted all internal interviews. The Consolidated Criteria for Reporting Qualitative Research (COREQ) guidelines were followed to ensure high-quality research design (File S1) [31].

### 2.6. Ethical Considerations

The Institutional Review Board at Ann and Robert H. Lurie Children’s Hospital of Chicago (IRB 2021-4077) determined that this study was exempt from IRB review on 20 October 2021. A study information sheet was provided to all participants during recruitment, and participants were notified that interviews were recorded. Participants were given an opportunity to review this manuscript prior to submission to ensure they felt their comments were appropriately de-identified and their ideas were accurately represented. An earlier version of this work was previously presented and published in abstract form [32].

## 3. Results

A total of 20 providers participated in the study, which included 8 nurse practitioners and 12 attendings (Table 1). Ten providers were internal to the PI’s institution, and there were ten external providers. In total, providers came from seven PICUs—two from the East Coast and five from the Midwest. Among the participants, 15 came from independent children’s hospitals and the rest from pediatric centers within larger adult institutions.

Four major themes were identified regarding PICU providers’ experiences in caring for CMC with NI: (1) distinct and growing population, (2) challenging traditional conceptions of PICUs, (3) ambivalence, and (4) adapting to find meaning. Themes, with supporting quotations, are further detailed in Table 2, Table 3 and Table 4.

### 3.1. Distinct and Growing Population

CMC with NI are a growing and distinct population in the eyes of respondents. This group of children was a distinct subcategory to all PICU providers interviewed. They were able to speak extensively about impactful experiences and emotions associated with CMC with NI. Many providers noted a subjective sense of increasing prevalence and/or degree of complexity of this population: “Kids who used to, you know, die all the time when I first started are now surviving with really chronic problems, and I think these patients are becoming more common” (MD/DO2).

Challenging Traditional Conceptions: CMC challenge traditional perceptions of the work of critical care providers. Providers expressed that the work of critical care that they chose, trained for, and were equipped to provide was generally considered the active resuscitation of acutely ill, previously well children. They distinguished this from the work of caring for CMC with NI, which was described as slow, repetitive, and detail-oriented. MD/DO6 explained, “They might not need a lot of [resuscitative] measures, but they need a lot of coordination, they need a lot of, you know, follow up outpatient appointments, which doesn’t necessarily fall into an intensive care domain”. Important contrasting viewpoints included a provider who reframed CMC with NI as “just as sick”, (NP2), and some providers who noted a particular interest in or affinity for these patients. MD/DO4 describes their work caring for this population with both genuine caring and a sense of obligation: “The cultural norm I observe in our unit is we’re good people and we care about these kids and families because we’re told to. I mean I do care about them, but I don’t know that we’re the best team, but we’ve been nominated and appointed to be their team”. Further quotes for themes 1 and 2 are in Table 2.

**Table 2:** Supporting quotes for themes 1 and 2.

Theme	Subtheme	Quotation
CMC are a growing and distinct population in the eyes of providers	Increasing prevalence and complexity	“When I did my first PICU rotation as a second year resident … [maybe] 10 to 20% of our patients on any given day were children with medical complexity and not even … years later, I would say it’s close to half” (MD/DO7).
CMC with NI challenge the traditional perception of the work of critical care providers and units	Caring for CMC is a different kind of work than “saving” previously healthy children	“I think some people who work in intensive care kind of seek the high of the rescue and sometimes caring for [CMC] doesn’t really fit that mold. This like daily grind of feeding intolerance, or ventilator setting tweaks, or growing, it is not as exciting as, you know, canulating someone on to ECMO” (NP3).
		“I think the most stressful thing is having a unit full of these kids, and knowing that there are times where we turn down transfers of kids that we potentially could save” (NP7).
	Some providers have a special affinity for CMC	“As someone who really generally really enjoys taking care of this population and, you know, even the reason that I went into medicine and pediatrics were some kids that I worked with [before] that kind of fell in the medical complexity area, it’s a population that is very near and dear to me” (MD/DO7).
	When CMC with NI do require intense resuscitation, some providers feel ambivalent	“I think I’ve noticed in myself and in the nurses and a lot of people there’s a question of almost like an underlying like ‘what’s the point?’ sort of question. You know pouring a lot of resources and time [for] someone who doesn’t seem like they’ll, let’s say, contribute to society in a meaningful way or experience life in a way that is ostensibly meaningful to us. And so, but … how you, any one of us interprets that is completely determined by what we value in life, what we think makes life meaningful and worth living. And so, not that it’s unimportant and it’s just not, it’s just generally not that relevant to any decisions we should be making about taking care of patients” (MD/DO9).

### 3.2. Ambivalence

Many of the interviewed PICU providers described ambivalence and mixed feelings about their experience caring for CMC with NI. Providers held a substantial amount of tension in their views about CMC with NI, and expressed both positive and negative experiences in caring for them. Some providers described the spectrum of viewpoints they witness among their colleagues. Others expressed their own ambivalence indirectly by describing both positive and negative emotions or viewpoints within their interview. One source of ambivalence was when CMC with NI can sometimes become severely ill and require intensive interventions. Providers worried that by providing this care they were causing harm due to unjust distribution of healthcare resources, perpetuating a child’s perceived poor quality of life and/or suffering, and/or adding to family and caregiver burdens. MD/DO12 summarized most of these concerns, noting they worry that “we’re causing harm. That we’re causing harm to—to patients, that we’re causing harm to their families, their siblings to, you know, to some degree to the system itself”. Other examples of ambivalence and mixed emotions (both positive and negative) are found in Table 3.

**Table 3:** Supporting quotes for theme 3.

Theme	Subtheme	Quotation
Providers feel ambivalent about CMC with NI	Providers describe varied emotions and perspectives about CMC with NI among their peers	“You know I definitely think these kids get treated differently … I can honestly say that you know nurses treat all patients with respect and dignity and they take care of them, but I do think there is [an] undercurrent you know, then it’s like ‘oh, [they] are [a] chronic kid and oh of course they’re sick’, and you know just kind of like a, ‘oh we expect this, oh, you know’, I think it can go as extreme as like, you know, people don’t maybe put as much thought or care in into those patients. I hope that’s not the case very frequently, but I think there can be some, I guess a good way to say would be like ‘burnout’ with those patients” (MD/DO5). “You know that can definitely happen, but I also see you know these kids get better and us get very excited that they’re getting better, or you know even in these CMC with really bad NI, us still you know walking by the rooms and [saying], ‘oh aren’t you a cute little baby’ and then cuddle and you know, so I think that they’re still loved there” (MD/DO5).
		“I think there’s a really broad spectrum of perspectives. Some of the people that work in our ICU are incredibly compassionate and open minded, look at these kids as you would a neurotypical child, and are really strong advocates for the families in terms of trying to get more access and resources at home. I think there are other people in our group, particularly physicians I don’t see it with nurse practitioners, physicians who typically, to them these children aren’t whole, that they’re not worth investing as much time and energy in, that their life isn’t worth as much and so they don’t care as much about the outcome. And it’s just kind of check the boxes and move them forward and get them back to their baseline and send them home” (MD/DO1).
	Provider emotional responses can be complex and multifaceted—here, a provider describes a single patient/family dyad as a cause of moral distress as well as a valued relationship	“I think people don’t understand what quality of life [Patient] has. … He basically just lays in the bed with no interaction, he’s not able to interact with his outside world, from the from a provider standpoint that’s what I see, and so I think people think then we just keep saving him, and saving him and saving him from these, you know, acute on chronic bouts of respiratory failure … so at what point do we say you know, ‘enough is enough’, in a sense, like are we torturing him?” (NP8).
		“I think she has a lot of trust in our team. But I think a lot of that, I think a lot of that trust is because we just have a good rapport. Like [inaudible] you know we don’t just talk about the plan of care for the day and the numbers and things like that we talk about you know she’s- she’s been in our system for so long, you know, she had … other kids in that timeframe and so it’s like ‘how the babies doing’, and things like that, you know we, we see her other children grow up, … I think we’re invested in her and [Patient] and their entire family because we know the entire family” (NP8).

### 3.3. Adapting to Find Meaning

Nevertheless, many providers still found positive meaning in this work by focusing on families and, to a lesser extent, the children themselves. Most providers reported a sense of purpose connecting with families over prolonged or repeated admissions. Most providers also reported satisfaction in helping families achieve their goals in progressing a child’s care. Every single provider used one or both strategies to create meaning: “I guess the best part is when family is set up for success to take care of their child in the way that they want to take care of their child” (NP4). Providers also enjoyed moments of connection with more interactive children. The ability of a child to interact, particularly to smile, was used by most providers to describe a patient and seemed to be an important factor in positive meaning-making for many, although not all. Most providers also found meaning in knowing they respected a family’s goals of care, while also stating that they would not choose life prolonging therapies for a family member of their own in a similar situation. Some providers seemed at peace with this distinction, but one provider noted a cumulative negative effect: “So I try not to think about it, and I definitely don’t allow that to affect how I care for these kids. But it does eat away [at] you a little bit, you know, day after day, doing things that you don’t just agree with 100%” (NP7).

**Table 4:** Supporting quotes for theme 4.

Theme	Subtheme	Quotation
Providers find adaptive meaning-making strategies when caring for CMC with NI.	Connecting with families	“We have one family that has come to our hospital for a very long time … they’re very near and dear, obviously, to us because we’ve known them since they were … growing up … That family was really, you know, a memory for us because we’ve walked through so many decisions with them” (MD/DO10).
	Empowering and serving families	“They teach us sort of how important it is to facilitate, uh, patients being sort of within the context of their family, and—and what that means for them and their family. Um, I think the most rewarding part is—is that—is sort of facilitating the ability for a child to go home with their family, um, uh, and making that happen for the family and the child”, (NP6). “I think constantly in critical care people have this, um, I don’t know, very arrogant, boastful approach … ‘We save lives’, and, ‘We do all this’—it isn’t about that. I don’t know about that, I think helping a family or a patient that’s complex like this take the steps that they can take and walk the path that they want to walk—I think that’s very rewarding” (MD/DO3).
	Family’s goals more important than provider opinions	“I just have to remind myself that you know, what the family, the family’s goals are might be different than mine, but that that is not my, you know, personal space to have, and so really I need to just continue to partner with the families and hopefully get the child back to the state that is the family’s expectation” (NP4).
	Interaction with children	“…those moments of them returning more to baseline and potentially like getting a smile or getting some sort of recognition or [an] int- you know, interaction with them and the families saying, ‘Oh, this is like much more like who they are’” (MD/DO12).
	Providers have complex thoughts that sometimes evolve	“I would say that there are some providers who really enjoy taking care of these kids and others, not so much, right, it varies, you know I mean, I would say that even in my own career, you know I probably started off, you know, not necessarily enjoying caring for these patients, because it can be a lot of work right. And then … just something that I, that I have been doing more of and enjoyed more as I’ve gotten older” (MD/DO2).

## 4. Discussion

In this qualitative study examining the perspectives of a group of PICU providers about their care of CMC with NI, themes were identified that describe providers’ perception of the increasing prevalence of CMC with NI in the PICU, how these patients’ unique needs challenge the traditional understanding of critical care medicine, the ambivalent relationship many PICU providers have with this population, and the strategies that can help providers adapt to and find meaning in this work. These findings mirror many of those in the literature with an important distinction between populations; while Graham et al. interviewed the parents of PICU patients with “severe antecedent disabilities”, the remainder of the noted qualitative studies focused on chronically critically ill children without mention of presence or absence of neurologic impairment [15]. This study directly explores NI as a potentially important cofactor in provider experiences and attitudes. In addition to the novel focus on NI, this study also adds additional emphasis on provider adaptive strategies and the role of cognitive dissonance. Both findings have been touched on but not fully developed in the prior literature.

The first two themes of “distinct and growing population” and “challenging traditional conceptions” demonstrates that CMC with NI were unique in the minds of PICU providers. The care of these children was viewed by some PICU providers as different from patients requiring “true” critical care, which supports findings of prior studies [15,19,22]. These findings suggest that, from the provider perspective, CMC with NI can be understood as the “wrong type of PICU patients” when they are “well”, but also the “wrong type of PICU patients” when they are “sick.” Either they do not require the intensive resuscitation providers were trained to perform, or they do require aggressive care but providers question the value of such interventions. Families of CMC in the Ames et al. study reported experiencing clinician apathy toward and dehumanization of their children; perhaps those experiences are driven by the clinician perspectives found in this study [8]. In the PICU, CMC with NI could be vulnerable to sub-optimal care due to providers’ possible subconscious bias as they manage a busy unit. Although actual care provision was beyond the scope of this study, this work suggests the need for more research into how provider attitudes and biases impact the care decisions they make.

The third theme explored the ambivalence that providers may feel about being tasked with this mismatched care. Ambivalence also affects some providers when they need to administer intensive resuscitation to CMC with NI who do become critically ill. These findings support and expand upon the existing literature concerning providers caring for children with CCI [19,20,21,22,23]. The variation in providers’ experiences is worth noting. Some of the providers interviewed had predominantly negative memories and opinions about CMC with NI, while some had very positive stories and beliefs, and none were exclusively positive or negative.

The fourth theme, “adapting to find meaning”, showed how some PICU providers make a particular point to find meaning and purpose in their work caring for CMC with NI. The value of provider–family relationships has been previously described from both the provider and parent perspective [16,20]. This study identified more novel meaning-making strategies, including finding meaning in family service and empowerment, and appreciating moments of connection with the children themselves, when possible. Finally, this study found that most providers had to explicitly reconcile the cognitive dissonance of providing care they would not personally choose. This type of mismatch between family and provider goals has been reported [20,22,23]. However, this study describes this phenomenon in greater detail, and demonstrates how most providers work to redefine the goal of their work to be caring for families and respecting family wishes.

Finally, the strategies identified seem to be adaptive and may represent targets for intervention. Promoting continuity and family relationships (for example, through ongoing research into primary intensivists [33]), offering opportunities for providers to visualize the benefits they provide to a child and family (perhaps via post-discharge updates and photos from families and primary pediatricians), and promoting opportunities for connection with children may all impact provider well-being through enhanced sense of purpose. However, each strategy also has important limitations. While providers value sharing a smile or moment of interaction with a child, they seem to feel more distress in keeping the most severely impaired children alive. As much as providers enjoy and find meaning in rich family relationships, many also feel particularly challenged when family–provider relationships are difficult or strained. Rationalizing the cognitive dissonance of providing care they personally disagree with by focusing on honoring family goals may seem to help some providers cope, but it may also be a potential source of burnout. A scenario-based survey of PICU nurses and respiratory therapists found that participants who disagreed with a hypothetical family’s choice to pursue aggressive ICU care for their child with NI had significantly higher levels of distress [18]. This mirrors NP7′s prophetic worry about this work “[eating] away” at them. More research is needed to determine how to prepare PICU providers to care for CMC with NI in a way that feels sustainable and fulfilling.

This study has limitations. Recruitment methods did not allow for tracking non-participants. Recruitment was conducted through two main streams—half the cohort was “internal”, from the PI’s home institution, and half was “external”, from other children’s hospitals. It is possible that these two groups of providers had different motivations for participating; the internal group to support internal research, and the external group due to specific topical interest. In fact, several external participants explicitly volunteered a positive interest in this patient population. This sample represents academic PICUs but may not reflect PICUs from regions of the country not in the sample, or PICUs in smaller, community-based, private, or low-resource setting hospitals. Data were not collected on the infrastructure or resources available at the PICUs represented in this study; we were thus unable to draw conclusions about how those resources impact the provider experience of caring for CMC with NI. Finally, discussions of sensitive topics are vulnerable to social desirability bias.

## 5. Conclusions

In summary, this study supports findings of previously published studies regarding provider experience caring for CCI and demonstrates how they apply to a more specific population—CMC with NI. Both distressing perspectives and adaptive meaning-making strategies co-exist among different PICU providers and often within providers themselves. Further research is needed in several directions. First, a broader sample of providers from PICUs in rural, community-based, private, international, and resource-limited hospitals would help describe the ways in which the local practice context impacts PICU providers’ perspectives about CMC with NI. Second, research can focus on providers’ adaptive strategies for making their work meaningful despite the cognitive dissonance sometimes experienced when they care for this population. Understanding specific strategies providers can use to process distress and dissonance in a healthy manner could create targets for future interventions. Finally, further studies regarding CMC and CCI should attend to NI and explicitly address issues of ableism and its possible effects on care delivery.

This study raises the critical question of how pediatric intensive care can adapt to the growth of this patient population. It seems that the modern PICU will need to adapt to serve two roles: to “rescue” previously healthy children, and to serve as inpatient medical home for a uniquely vulnerable group of children. Rather than conceptualizing this shift as being imposed upon PICU providers who feel “nominated and appointed” for the task, it may be helpful to reframe this as the intentionally chosen future direction of the field.

## Figures and Tables

**Table 1:** Provider characteristics and demographics.

Provider Characteristics		Number of Providers (*n* = 20 ^1^)
Type of Provider	Nurse Practitioner	8 (40%)
	Attending Physician	12 (60%)
Race	Asian	2 (10%)
	Black/African American	1 (5%)
	White	17 (85%)
Years of Experience	1–5 years	3 (15%)
	5–10 years	8 (40%)
	10–20 years	6 (30%)
	20+ years	3 (15%)
Institutions	Internal	10 (50%)
	External ^2^	10 (50%)
Presence of a Complex Care Team	Yes	18 (90%)
	No	2 (90%)

^1^ Including the “internal” PICU, a total of 7 PICUs in 6 different states were represented. ^2^ In both cases where a provider answered “no” to the question, at least one other provider from the same institution answered “yes”, making it difficult to draw conclusions based on this data point.

## Data Availability

Participants did not give permission for full interview transcripts to be published; therefore, the data will not be made publicly available, but specific requests can be directed to the corresponding author for consideration.

## Supplementary Materials

The following supporting information can be downloaded at: https://www.mdpi.com/article/10.3390/children12010034/s1, File S1: COREQ checklist; File S2: Interview Guide.
