# Supplementary material for: Pediatric Intensive Care Provider Attitudes About Children with Medical Complexity and Neurologic Impairment: A Qualitative Study
Source: Children (Basel). 2024 Dec 28;12(1):34. doi: 10.3390/children12010034 (PMC11763489; doi:10.3390/children12010034)
Supplement: Supplementary file 1 [file children-12-00034-s001.zip › File S2.pdf]

Interview Guide

Note that the investigators amended the interview guide iteratively throughout data collection based on earlier interviews to emphasize emergent themes and ideas and enhance the quality of the interview content.

1. [Introduction, to be read aloud]
   a. During our discussion today, we want you to share your experiences and stories, as well as your opinions and feelings about caring for a group of children we will call "Children with medical complexity", or "CMC". In the PICU, we have many terms for these children and they are a heterogenous group, so I want to be sure we are talking about the same patients. For the purposes of this discussion, the patients we want to discuss with you are those that Dr. Eyal Cohen et al. have defined as "CMC". According to this definition, CMC must meet the following four criteria:
      i. They must have substantial family-identified healthcare service needs, including medical, therapy, or educational needs, that have a significant impact on their family.
      ii. At least one known or unknown chronic clinical condition associated with medical fragility (high morbidity or mortality).
      iii. Severe functional limitations which may or may not require technology to mitigate.
      iv. High healthcare resource utilization.
   b. I specifically want to focus on the children in this category who also have neurologic impairment, or NI. Berry et al. defines NI as "static and/or progressive, central and/or peripheral neurologic diagnoses associated with chronic functional and or/intellectual impairment." Therefore, a neurotypical child with cancer and a port will likely meet the definition of a CMC but is not the specific child I wish to discuss.
   c. As you can see, children with tracheostomies will meet this definition. However, many children without chronic airway or breathing support will also easily qualify as CMC with NI. I want you to think broadly about different children you've cared for who likely meet this definition as we talk today.
   d. The goal of our discussion today is to better understand the experiences of providers who care for CMC with NI in the PICU. Our hope is ultimately to improve both the quality of care these children experience when hospitalized in the PICU as well as improve the experience of the providers caring for them, and understanding that experience is the first step.
   e. This is a challenging topic and many providers may have difficult emotions or controversial opinions. I appreciate your

time and hope you will be willing to be as honest as possible in your responses. I may ask follow-up questions or shift the topic in order to cover all the topics in our time together.

f. Confidentiality and Recording: This interview is being recording and will be transcribed verbatim; the transcript will be edited to remove names or identifying information. You will remain linked to your institution in the analysis, but the name of the institution will be de-identified as well. By participating in this study you consent to being recorded. The recording and transcript will be securely stored. Your responses will be completely anonymous.

g. If you have additional thoughts, you are welcome to reach back out to me via email.

h. You will be sent a draft of the manuscript and given an opportunity to review it prior to submission for publication. This is to ensure that you feel your comments have been appropriately de-identified and to verify that your statements and ideas have been accurately represented.

2. Demographics to be gathered by pre-survey.

    a. Let's pause now to complete a brief demographic survey. [Interviewer will instruct the interviewee how to access the Qualtrics survey on their device. A few minutes will be allowed for them to fill it out.]

3. Questions—sub-bullets are probes which may be used as indicated.

    a. Can you tell me about a memorable patient encounter with a CMC with NI?

        i. What were your interactions like with this child? With their parents?
        ii. Describe the child's neurologic status.
        iii. Did you feel like the child was in pain? Delirious? Agitated? Aware of their hospitalization? More or less comfortable than you suspect they were at home?
        iv. Did you feel that you understood what the clinical problem was in this situation, or the reason for admission? Did you feel like you understood what your goal was?
        v. Is there anything else you can think of which made this a memorable positive experience for you as this child's provider?

    b. Can you tell me about a patient encounter with a CMC with NI in the PICU which you remember as a [opposite] experience?

        i. What were your interactions like with this child? With their parents?
        ii. Describe the child's neurologic status.
        iii. Did you feel like the child was in pain? Delirious? Agitated? Aware of their hospitalization? More or less comfortable than you suspect they were at home?

iv. Did you feel that you understood what the clinical problem was in this situation, or the reason for admission? Did you feel like you understood what your goal was?
v. Is there anything else you can think of which made this a memorable negative experience for you as this child's provider?

c. Can you tell me a time when there was disagreement or tension among the medical team about the care of a CMC with NI in the PICU?
   i. Do you feel like there are opportunities to discuss these things as a group? In what context—formal or informal?
   ii. Was that unusual or is that a pattern you sometimes see playing out?
   iii. How does attending handoff/variability affect these kinds of decisions?
d. What are some of the terms you or your team use to describe these patients?
   i. Are there some terms that some people use and some people don't?
e. What, if anything, have you seen change in the care of this population over time?
f. What are some of the best or most rewarding parts about your job taking care of CMC with NI in the PICU?
g. What are some of the worst or most frustrating parts about your job taking care of CMC with NI in the PICU?
h. What kinds of attitudes or perspectives do you notice among your peers and colleagues towards caring for CMC? Do you feel like they are similar to yours or different?
i. What do you worry about the most when you think about your patients with CMC and NI?
j. Do you have any other thoughts that come to mind on this topic that we haven't discussed yet? Anything else you feel like I ought to know?
